# A New Way to Use Hydrophobic Deep Eutectic Solvents: Improved Lead Detection Using an Ion‐Selective Electrode with a Polymer Membrane Modified by them

**DOI:** 10.1002/cphc.202500500

**Published:** 2025-09-28

**Authors:** Cecylia Wardak, Malgorzata Grabarczyk, Mersiha Suljkanović, Jasmin Suljagić, Magdalena Wardak

**Affiliations:** ^1^ Department of Analytical Chemistry, Institute of Chemical Sciences, Faculty of Chemistry Maria Curie‐Sklodowska University in Lublin Maria Curie‐Sklodowska Sq. 3 20‐031 Lublin Poland; ^2^ Department of Chemistry, Faculty of Natural Sciences and Mathematic University of Tuzla Urfeta Vejzagića 4 75000 Tuzla Bosnia and Herzegovina; ^3^ Department of Organic Chemistry, Faculty of Technology University of Tuzla Urfeta Vejzagića 8 75000 Tuzla Bosnia and Herzegovina; ^4^ Independent Public Health Care Center of the Ministry of Internal Affairs and Admin‐istration in Lublin Grenadierów St.3 20‐331 Lublin Poland

**Keywords:** hydrophobic deep eutectic solvents, ion‐selective electrodes, lead determination, organic matrix, potentiometry

## Abstract

In this study, the use of terpene‐based hydrophobic deep eutectic solvents (HDESs) in the preparation of polymeric membrane ion‐selective electrodes is presented. HDES obtained from terpenes (menthol and thymol) and octanoic acid are used as a new component of polymeric membrane of potentiometric sensors sensitive to lead ions. Electrodes containing different amounts of HDES in the membrane (from 1 to 12 % wt./wt.) are prepared, and potentiometric measurements are carried out for these electrodes to determine the detection limit, the slope of the characteristic, and the response linear range. Based on the analysis of electrode performance, it is found that the optimum concentration of HDES in the membrane is 5 wt%. For such membranes, a more detailed study is carried out using a solid contact sensor. Selectivity toward interfering species as well as potential stability and reversibility, optimum pH range, effect of light, and presence of gases in the sample solution are investigated for such sensors. The obtained measurement results indicate that the tested sensor containing HDES in the membrane has good analytical parameters, and excellent selectivity (log K ≤ −4.4). It has been successfully used to determine lead in real environmental water samples after a brief pretreatment with XAD‐7 resin.

## Graphical abstract

First use of hydrophobic deep eutectic solvents (HDESs) in the preparation of polymeric membrane lead‐sensitive ion‐selective electrodes is presented. It is found that enrichment of the membrane with HDES additive improves the electrode's parameters, primarily lowering the detection limit and increasing selectivity. The proposed electrodehas been successfully used to the analyse real samples.

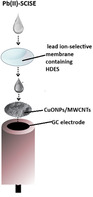

## 1 Introduction

Since the term “deep eutectic solvent” (DES) first appeared in the scientific literature two decades ago,^[^ ^1^ ^]^ stating that new designed solvents could meet the principles of “green” chemistry, it attracted great attention of researchers. DESs were acknowledged as a “new” class of ionic liquids (ILs), due to their similar physical properties, although their chemical properties suggest different application areas. Over the years, research has been focused on the use of DESs as alternative media for metals that are traditionally difficult to plate or process or involve environmentally hazardous processes.^[^ ^2^ ^]^ Many researchers were preparing new DESs from a wide range of starting components, in simple preparation procedures that included gentle mixing of solid components, with or even without heating, resulting in transparent liquid which do not require further purification, contrary to ILs^[^ ^3^ ^]^. After preparation, fourier transform infrared spectroscopy (FTIR) and NMR spectral analysis follows, in order to confirm hydrogen‐bonding interactions between DES components, as well as determination of their physicochemical properties (density, viscosity, thermal stability, and water content) and finally application for certain analytical purposes. However, most of the DES‐related studies included hydrophilic DESs, and the first studies involving hydrophobic DESs (HDESs) that appeared in 2015 established four criteria to assess the usability of HDESs: low viscosity, high hydrophobicity, density different from water, and minimal influence of pH.^[^ ^4^ ^]^ Hydrophobicity of these solvents makes them attractive as promising alternatives to conventional organic solvents in sample preparation and liquid–liquid extraction (LLE) of nonpolar analytes and transition metals. From the first application of HDESs for metal ion removal from nonbuffered water solutions, it was obvious that maximum of extraction efficiency can be obtained after very short equilibrium time (5 s), and authors suggested that ion‐exchange principle was the driving force for this process, proposing that positive metal ions were replaced with partially positive lidocaine (HDES was prepared from lidocaine and decanoic acid).^[^ ^5^ ^]^ Many researches also confirmed successful application of HDESs as suitable extractants for metal ions, with desirable properties such as low viscosities and high hydrophobicities, resulting with high removal rate in simple, fast, and low‐cost (low solvent consumption) procedure, which did not require any additional ligand as a carrier.^[^ ^6^, ^7^, ^8^, ^9^ ^]^ Recent studies have shown that DESs have immense potential as green substitutes for commonly used toxic organic solvents.^[^ ^10^, ^11^ ^]^

Considering high selectivity of tested solvents for Pb(II) ions,^[^ ^8^, ^9^ ^]^ the challenge for further study of DES applications in the field of electrochemical sensors arose. For electrochemical sensors and biosensors, the main use of DESs has been in procedures for modifying electrode surfaces: the high viscosity of the DES altering the rate of modification, principally owing to the reduced rate of diffusion;^[^ ^12^ ^]^ this influences the active surface area, the surface morphology, and electrocatalytic effects, compared to the same protocols carried out in aqueous electrolyte solution. Previously published results confirmed enhanced analytical sensing parameters, besides improving the “green” characteristics.^[^ ^13^ ^]^

Polymer membrane ion‐selective electrodes (ISEs) are the most commonly used and studied potentiometric sensors. In the case of such electrodes, the mechanisms of the processes responsible for membrane potential formation are extraction and ion exchange.^[^ ^14^, ^15^ ^]^ The performance of the membrane in selective ion recognition, and thus, the electrode parameters depend primarily not only on the ionophore but also on other components affecting the extraction capacity of the membrane.^[^ ^16^, ^17^ ^]^ Therefore, given the very high efficiency in the LLE of lead ions using terpene‐based HDESs, we decided to use them as components of polymeric electrode membranes sensitive to Pb(II) ions.

Scientists’ interest in developing sensors for lead detection has remained strong for many years. In the last 5 years alone, dozens of articles devoted to lead ISEs have been published. One of the research directions in this area concerns new active substances that can act as ionophores in membranes of electrodes sensitive to lead ions. Among the proposed ionophores are, among others, [2‐{4‐(diphenylphosphanyl) phen‐2‐Yl} pyrimidin‐4‐Yl]‐3‐(triethoxysilyl) prop‐1‐Yl urea,^[^ ^18^ ^]^ coumarin derivatives,^[^ ^19^ ^]^ [2‐(1‐(4‐(3‐(4‐chlorophenyl)ureido)phenyl)ethylidene)hydrazine carbothioamide,^[^ ^20^ ^]^ hydrazinecarbothioamide derivative,^[^ ^21^ ^]^ and polycarbazole Sn(iv) arsenotungstate nanocomposite (NC).^[^ ^22^ ^]^ Another area of research involves work on electrode design and focuses primarily on new solid contact materials that allow for the creation of high‐performance electrodes without an internal electrolyte solution. Recently, many new nanomaterials, including nano vanadium pentoxide nanoparticles,^[^ ^19^ ^]^ disordered mesoporous carbon,^[^ ^23^ ^]^titanium carbide Ti_3_C_2_,^[^ ^24^ ^]^ and NCs including polyaniline nanowires and MXene nanosheet composite,^[^ ^25^ ^]^ tin oxide–tin(IV) antimonophosphate (SnO_2_/Sn(IV)SbP) NC,^[^ ^26^ ^]^ polyaniline/montmorillonite composites,^[^ ^27^ ^]^ carbon nanofibers, and IL NC,^[^ ^28^ ^]^ have been successfully used as solid contact^[^ ^27^ ^]^ in Pb(II)–ISEs. The best electrodes developed to date have shown a detection limit of 10^−8^ mol L^−1^. Although this is a promising value, the solutions to further reduce the limit of detection while maintaining high selectivity and stability of the electrode readings are still being sought.

In this work, we propose a new ecofriendly approach to Pb–ISE construction using HDES in classic electrodes and in all‐solid‐state electrodes. To the best of our knowledge, these compounds have not yet been used in the preparation of polymeric membrane ISEs.

## 2 Results and Discussion

In this article, lead ISEs with polymeric membrane doped with terpene‐based HDES were studied. Two kinds of HDES composed of menthol or thymol and octanoic acid were used. The HDES content in the membrane varied from 1% to 12% wt./wt. As a control system, an electrode with a membrane of classical composition without the addition of HDES was tested in parallel.

### 2.1 Effect of HDES Content on Potentiometric Electrode Response

**Figure 1:**
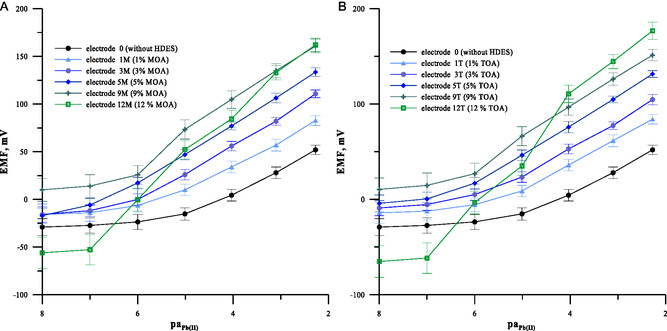
Calibration curves of lead electrodes with different contents of HDES in the membrane; electrodes based on A) MOA and B) TOA. Error bars show the standard deviation for three identical electrodes (*n *= 3).

The potentiometric response of studied electrodes was studied by potential measurements in Pb(NO_3_)_2_ solution in the concentration range 1 × 10^−2^–1 × 10^−8^ mol L^−1^. The obtained calibration curves are shown in **Figure** **1** for electrodes containing MOA in the membrane phase (1A) and for electrodes containing TOA in the membrane phase (1B). As shown, membrane modification by the addition of HDES caused a change in the electrode response in both cases. On the basis of the obtained calibration curves, the analytical parameters of particular electrodes such as the limit of detection, the working concentration range, and the characteristic slope are determined and summarized in **Table** **1**. As expected, enrichment of the ion‐selective membrane (ISM) with HDES affects its potentiometric response. As the HDES concentration increases, the sensitivity of the electrode increases and the linearity range of the calibration curve lengthens, thereby lowering the detection limit. This trend continues up to 5% HDES in the membrane. For higher concentrations of 9% and 12% wt., a deviation from the straightness of the calibration curve with a super‐Nernstian slope is observed in the range of lower lead concentrations. This was observed for both compounds tested, with the effect being stronger for MOA. On the basis of the results obtained, the optimum content of HDES in the membrane was set at 5% wt. Electrodes with membranes containing this amount of HDES showed the best performance with a detection limit of 6.28 × 10^−8^ and 2.18 × 10^−7^ mol L^−1^, a linearity range of 1 × 10^−2^–1 × 10^−7^ and 1 × 10^−2^–1 × 10^−6^ mol L^−1^, and a characteristic slope of 29.83 and 28.68 mV pa^−1^ for the 5 MOA and 5 TOA electrodes, respectively. Comparing the two HDESs tested, it can be seen that the MOA‐based electrode shows better performance; therefore, this membrane composition was used in further studies.

**Table 1:** Analytical parameters of lead ISEs with different contents of HDES in the membranes.

Electrode	Slope of characteristic [mV/Pa]	LOD [mol L^−1^]	Liner range [mol L^−1^]
Electrode 0	27.01	6.30 × 10^−6^	1 × 10^−2^–1 × 10^−5^
Electrode 1M	27.67	2.40 × 10^−6^	1 × 10^−2^–1 × 10^−5^
Electrode 3M	28.37	5.82 × 10^−7^	1 × 10^−2^–1 × 10^−6^
Electrode 5M	29.83	6.28 × 10^−8^	1 × 10^−2^–1 × 10^−7^
Electrode 9M	31.80	3.40 × 10^−6^	1 × 10^−2^–1 × 10^−5^
Electrode 12M	–	–	No linear response
Electrode 1T	27.33	1.90 × 10^−6^	1 × 10^−2^–1 × 10^−5^
Electrode 3T	28.30	3.54 × 10^−7^	1 × 10^−2^–1 × 10^−6^
Electrode 5T	28.68	2.18 × 10^−7^	1 × 10^−2^–1 × 10^−6^
Electrode 9T	30.91	1.44 × 10^−6^	1 × 10^−2^–1 × 10^−5^
Electrode 12T	38.9	6.58 × 10^−5^	1 × 10^−2^–1 × 10^−4^

### 2.2 Solid Contact ISE

In recent years, electrodes that do not contain an internal electrolyte in their structure, called solid contact ISEs (SCISEs), have been successfully introduced into analytical practice. Compared to classical designs, they are much more convenient to use, easy to miniaturize, and cheaper to manufacture and, in addition, can operate in any position and are more resistant to damage. Since SCISEs perform much better in environmental analyses, this type of electrode was further investigated in detail with a membrane of optimum composition (5% MOA). In the case of solid contact‐type electrodes, a suitable electroactive material called a solid contact is needed to ensure an efficient charge transfer process between the polymeric ISM and the electrode substrate. So far, different materials were used including conducting polymers, carbon‐based nanomaterials, metals, and metal oxide nanoparticles. Recently, it has been shown that in many sensor applications, it is even better to use composite or hybrid materials that exhibit more favorable properties than their component parts, for example, better electrocatalytic properties, higher hydrophobicity, and higher electrical capacitance, which results in more favorable performance of sensors based on these composites.^[^ ^26^, ^29^ ^]^ A promising solid contact material is a composite of multiwalled carbon nanotubes (MWCNTs) and copper oxide nanoparticles. It exhibits mixed ion–electron conductivity, a very high specific surface area, a large electrical capacitance, and a hydrophobic character. We have confirmed its usefulness in the construction of SCISEs in previous work.^[^ ^30^, ^31^ ^]^ It has also been used in this work to construct SCISEs with a membrane containing 5 wt% MOA (denoted hereafter as the 5MSC electrode).

**Figure 2:**
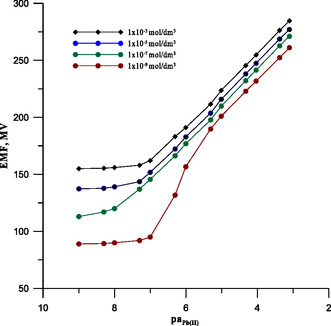
Calibration curves of electrode 5MSC conditioned in lead solutions of different concentrations.

For SCISEs, especially those designed for low concentrations, it is important to optimize the concentration of the conditioning solution, as this significantly affects the detection limit of the electrode. Therefore, we started our measurements with the electrode 5MSC by investigating the influence of the composition of the conditioning solution on the response of this electrode. The calibration curves of 5MSC electrodes conditioned in lead ion solution at different concentrations are shown in **Figure** **2**. As shown, the electrode's detection limit decreases with the dilution of the conditioning solution. This effect occurs up to a concentration of 1 × 10^−7^ mol L^−1^. Further dilution of the conditioning solution did not produce a positive effect. An electrode conditioned in a solution with a lower lead concentration (1 × 10^−9^ mol L^−1^) showed a nonlinear response at lower sample concentrations. The 5MSC electrode showed the best performance after conditioning in a 1 × 10^−7^ mol L^−1^ lead ion solution, showing a detection limit of 5.3 × 10^−9^ mol L^−1^, which was an order of magnitude lower than that of an electrode conditioned in a classical conditioning solution. It is worth noting that the electrode response was fully repeatable. The relative standard deviation from five repeated measurements for limit of detection was 3.1% and for the slope of the characteristic curve was 1.3%.

### 2.3 Selectivity

Selectivity is among the most important parameters of ISEs. This parameter was estimated from the values of selectivity coefficients, which were determined for 0, 5M, and 5MSC electrodes using the separate solution method. The obtained values of the selectivity coefficients in logarithmic form are presented in **Table** **2**, where it can be observed that modification of the ISM with the addition of MOA results in improvement of the membrane selectivity (lower values of log K). This improvement in selectivity is due to the increased extraction properties of the membrane toward lead ions as a result of the enrichment of its composition with MOA. Comparing the selectivity of electrodes with a membrane containing 5% MOA with a liquid and a solid contact, it is evident that the type of contact has no effect on the selectivity of the electrode, which is determined primarily by the composition of the membrane.

**Table 2:** Comparison of the selectivity coefficient values for electrode 0, electrode 5M, and electrode 5MSC.

Ion	Log K^pot^_Pb(II)/M_		
	Electrode 0	Electrode 5M	Electrode 5MSC
Cd^2+^	−3.2	−4.9	−4.9
Cu^2+^	−3.1	−4.5	−4.4
Ba^2+^	−4.8	−6.4	−6.5
Ca^2+^	−4.9	−6.9	−6.8
Mg^2+^	−5.1	−7.8	−7.8
Zn^2+^	−5.8	−6.0	−6.1
Co^2+^	−5.9	−6.4	−6.4
Ni^2+^	−6.0	−6.4	−6.5
Na^+^	−4.4	−7.0	−7.0
K^+^	−4.7	−7.3	−7.2
Li^+^	−4.9	−7.1	−7.0

### 2.4 Dependence of Electrode Potential on pH

For the practical application of the electrode, it is important to know the pH range of the sample in which the electrode potential is constant and does not depend on pH. Dependence of the potential of electrode 5MSC on pH was investigated in the 1 × 10^−4^ mol L^−1^ Pb(NO_3_)_2_ solution over pH range 2.0–8.0. The pH of the sample solution was adjusted using universal buffer solutions, and the working pH range was determined where the potential of the electrode was almost constant (±1 mV). Based on these measurements, the working pH range is 3.0–7.0. Beyond this range, a gradual change in electrode potential was detected. At higher pH values, decrease of the potential was observed. It was connected with the formation of hydroxy complexes of Pb^2+^ ions. Below pH 3, a decrease in electrode potential was also observed. In this case, it is due to protonation of the ionophore in the membrane phase.

### 2.5 Potential Stability and Reversibility

**Figure 3:**
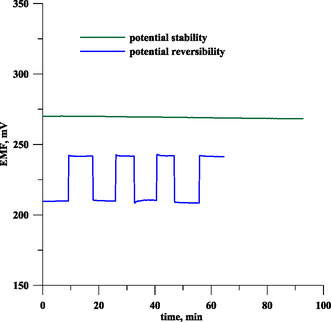
Potential stability and reversibility of a 5MSC electrode.

SCISEs often suffer from problems of stability and potential reversibility. These problems always occur for electrodes of the coated wire type, in which the ISM is in direct contact with the substrate electrode. For the electrodes investigated in this study, an intermediate layer in the form of a CuONPs/MWCNT NC was used to ensure good stability of electrode readings. In order to check the potential stability of the 5MSC electrode, the electrode was placed in a lead solution of 1 × 10^−3^ mol dm^−3^, and the potential was measured over a period of 90 min (**Figure** **3** green line). From the obtained potential measurements, the potential drift was determined as ΔSEM/Δ*t*, which was very low at only 0.021 mV min^−1^.

The potential reversibility of the 5MSC electrode was determined by measuring its potential over time in alternating lead ion solutions of 1 × 10^−4^ and 1×10^−5^ mol L^−1^. The measure of the reversibility of the electrode potential is the standard deviation from the mean value of the electrode potential for a given concentration. The time course of the potential variation of the 5MSC electrode during this experiment is shown in Figure 3 (blue line). The mean potential values were 241.56 ± 0.26 and 209.58 ± 0.80 mV for concentrations of 1 × 10^−4^ and 1 × 10^−5^ mol L^−1^, respectively. The low values of the standard deviation indicate the very good reversibility of the electrode potential.

### 2.6 Effect of Light and Presence of Gases

All solid‐state electrodes are sometimes sensitive to changing light conditions and the presence of gases such as O_2_ or CO_2_ in the sample. This sensitivity manifests itself in the variability of the electrode potential depending on the measurement conditions. In order to estimate the sensitivity of the tested electrode to the presence of gases in the sample and variable light intensity, a series of measurements were performed in a 1 × 10^−4^ mol L^−1^ Pb(NO_3_)_2_ solution, alternately for a solution saturated with O_2_ and CO_2_ gases and for a solution through which N_2_ was passed for 20 min (in order to remove the previous gases).

Similarly, the effect of light was tested. In this case, measurements were performed alternately in full daylight and in darkness. The average potential value obtained from five measurements in gas (O_2_ and CO_2_)‐saturated solution was 241.4 ± 0.8 mV, and in a nitrogen‐saturated solution, it was 241.9 ± 0.6 mV. Similarly, the average values of the potential measured for the electrode in light and darkness were 241.2 ± 1.1 and 242.1 ± 0.9 mV, respectively. Very small differences in electrode potential values (less than 1 mV) measured under different conditions and low standard deviation values prove that the tested 5MSC electrode is insensitive to changing measurement conditions. This is a valuable property in the context of its potential application for the analysis of real samples.

### 2.7 Effect of Real Sample Organic Matrix

Due to the instability of the potential during the analysis of river water samples, it was decided to investigate precisely how the organic matrix of such samples can affect the signal stability of the developed sensor. Among the main components of the organic matrix of aqueous environmental samples, we can distinguish surfactants and humic substances.^[^ ^32^, ^33^, ^34^, ^35^ ^]^ Surfactants enter the environment mainly as a result of human activities related to industry and agriculture. These include the production not only of cosmetics, cleaning products, and pharmaceuticals but also of food, textile, metallurgical, and many other industries. Their use is due to their unique properties to achieve the desired effects, such as foaming, dissolving, emulsifying, or dispersing.^[^ ^36^, ^37^ ^]^ Unfortunately, these properties can interfere with the stability of potentiometric sensor readings. Unlike surfactants, which enter environmental waters as a result of human activity, humic substances are naturally occurring organic compounds, formed by the decomposition of organic matter. On the one hand, they enrich soils and waters with organic matter; on the other hand, they can contribute to environmental pollution^[^ ^38^, ^39^ ^]^ and, in our case, affect the stability of the sensor potential.^[^ ^40^ ^]^ Therefore, the effect of different surfactants and humic substances on the signal of the proposed sensor was precisely investigated. The following compounds were selected for the study: CTAB, cationic surfactant; SDS, anionic surfactant; Triton X‐100, nonionic surfactant; rhamnolipid, biosurfactant; HA, humic acids; FA, fulvic acids; and NOM, natural organic matter. Such a wide range of organic compounds with different properties made it possible to investigate in detail how the potential components of organic matter present as a matrix of aqueous environmental samples can affect the signal stability and analytical parameters of the newly developed 5MSC electrode.

**Figure 4:**
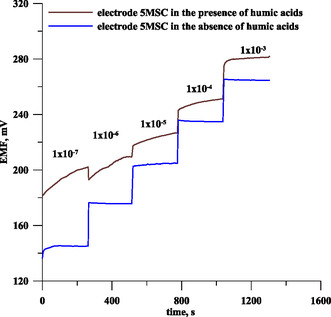
Potentiometric response of a 5MSC electrode versus time determined in Pb(NO_3_)2 solution in the absence and presence of 10 ppm humic substances.

The study consisted of comparing the sensor response for solutions containing and not containing the respective surfactants and humic substances. First, the sensor response was determined in lead solutions not containing the tested interferents. Then, increasing concentrations of Triton X‐100 were introduced into the standard lead solutions in the concentration range of 1–10 ppm, and the sensor performance was determined for each addition of Triton X‐100. In this way, it was possible to follow the changes in the potentiometric response of the tested sensor to Pb(II) ions in the presence of different concentrations of Triton X‐100. In an analogous manner, it was investigated how the sensor performance changes in the presence of different concentrations of SDS, CTAB, rhamnolipid, HA, FA, and NOM. Preliminary studies carried out allow us to conclude that humic substances interfere with measurements to a greater extent, since their lower concentration than surfactants causes a deterioration in sensor performance, primarily instability of the potential readings, an increase in the detection limit, and a decrease in the slope of the calibration curve. Among the humic substances, humic acids are the most interfering interferent, to a lesser extent NOM, and to the least extent HF. Among surfactants, CTAB is the most interfering surfactant and to a lesser extent Triton X‐100 and rhamnolipid, and SDS has the least negative effect on the analytical signal of the developed sensor. To eliminate these interferences, premixing of the sample with Amberlite XAD‐7 resin was used. The purpose of premixing with resin is to adsorb surfactants and humic substances contained in the sample onto the surface of the resin, so that their concentration in solution is reduced and thus there is less interference with the analytical sensor signal.^[^ ^41^, ^42^ ^]^ Based on preliminary tests, mixing with the resin for 5 min was chosen, using 0.5 g of resin per 10 mL of solution. Under these conditions, the performance of the sensor was once again tested in the presence of different concentrations of interferents. It was found that the introduction of a mixing step with Amberlite XAD‐7 resin allows a stable sensor signal to be obtained in the presence of up to 10 ppm humic substances and surfactants. An example of the potentiometric response of a 5MSC electrode in the presence and absence of humic substances is shown in **Figure** **4**. The tests carried out confirmed that mixing the sample with the resin results in more stable sensor signals, which in turn increases the sensor's sensitivity and range of linearity.

### 2.8 Analytical Application of the Electrode

The practical usefulness of the proposed 5MSC electrode has been demonstrated through its application to the determination of lead content in real samples, including tap water and river water. The tap water was analyzed without any pretreatment just after the addition of ionic strength buffer solution (0.001 mol L^−1^ Mg(NO_3_)_2_) and acetate buffer solution pH 5.0 (0.01 mol L^−1^). River water was additionally pretreated as described in the Experimental Section. The analysis was performed by the standard addition technique. Recovery tests were also carried out to verify the correct operation of the electrode. The results are summarized in **Table** **3** where it can be seen that the quantitative recovery was close to 100% and ranged between 96.8% and 103.7% which confirms that the electrode worked correctly. This fact was further confirmed by the successful use of the electrode to determine lead in a wastewater sample using certified reference material SPS‐WW1 Batch 113. The sewage sample was analyzed in the same way as the river water sample after being diluted with distilled water in a 1:1 ratio. The measured lead content was 97.9 ± 2.6 μg L^−1^, which was consistent with the certified value of 100 ± 0.5 μg L^−1^. Thus, the proposed electrode provides a good alternative for the environmental monitoring of lead.

**Table 3:** Results of the determination of Pb^2+^ by the proposed 5MSC electrode in real water samples.

Sample	Added Pb^2+^ [μg dm^−3^]	Found Pb^2+^, by ISE [μg dm^−3^]a)	Recovery [%]
Tap water	–	–	–
Tap water + 10 μg dm^−3^	10	9.82 ± 0.51	98.2
Tap water + 30 μg dm^−3^	30	31.11 ± 1.22	103.7
Tap water + 100 μg dm^−3^	100	98.3 ± 3.56	98.3
River water	–	13.22 ± 1.41	
River water + 10 μg dm^−3^	10	22.65 ± 1.63	102.5
River water + 30 μg dm^−3^	30	41.83 ± 2.35	96.8
River water + 100 μg dm^−3^	100	114.2 ± 4.88	100.8

a) Results are based on four measurements.

## 3 Conclusion

This article presents a new area of application for HDESs based on terpenes. These compounds effectively act as an additional component of a lead‐sensing ISM by increasing the ionic affinity for lead. A membrane electrode with an optimal HDES content of 5 wt% showed very good analytical performance both as a classical design and as a solid contact electrode. In addition, for SCISE, lowering the detection limit to 5.3 × 10^−9^ mol L^−1^ was achieved by optimizing the concentration of the conditioning solution. An effective way of eliminating the complex organic matrix of real samples was developed, which allowed the effective application of the proposed electrode to the analysis of real samples. The promising results obtained for the lead electrode are the starting point for the design of further electrodes sensitive to other heavy metal ions for which HDESs have proven to be good extractants. These include cadmium, copper, nickel, cobalt, iron, and others that have not yet been confirmed. Combined with the diversity of suitable solid contact materials and their versatility, this creates new opportunities for the design of new sensors as well as multisensor platforms.

## 4 Experimental Section

4.1

4.1.1

##### Reagents

For HDES preparation, solid starting components were used as follows: 1) As H‐bond acceptors (HBAs): a) L‐menthol (C_10_H_20_O, 99%, Sigma–Aldrich) and b) thymol (C_10_H_14_O, 99%, Sigma–Aldrich) were used. 2) As H‐bond donor (HBD): octanoic acid (C_8_H_16_O_2_, 99%, Acros Organics) was used.

Two HDESs were prepared by mixing HBA with HBD, both in 1:1 molar ratio of components. They were assigned as MOA and TOA for DES obtained from L‐octanoic acid with menthol and thymol, respectively. The formation of HDESs was studied using a standard procedure.^[^ ^43^ ^]^ The first component was weighed in the flask, while the second component was weighed separately and then transferred to the flask.^[^ ^44^ ^]^ After mixing, the solid components were melted at 40 °C until the resulting solvent was stable.^[^ ^45^ ^]^ Water content in HDESs was determined in a previous study,^[^ ^9^ ^]^ as well as their viscosity and density. The structures of the prepared solvents were confirmed earlier^[^ ^9^ ^]^ by FTIR spectra.

For membrane preparation beyond DES, the following components were used: tert‐butylcalix[4]arene‐tetracis(N,N‐dimethylthioacetamide) (lead ionophore IV) (Fluka), bis(1‐butylpentyl) adipate (BBPA) (Fluka), poly(vinyl chloride) (PVC) high molecular weight (Aldrich), and tetrahydrofuran (THF) (Chempur). NC used as solid contact was prepared from copper oxide nanoparticles (CuONPs) (particle size <50 nm and purity >99.5%) and MWCNTs (length: 3–6 um, outer diameter 10 ± 1 nm, and inner diameter 4.5 ± 0.5 nm). Both components were purchased from Sigma–Aldrich.

For pH adjustment, during electrode pH range determination, the following universal buffer solutions were used: for pH 2–3.8, buffer solutions were prepared by mixing the corresponding amounts of 0.02 mol L^−1^ potassium phthalate monobasic (with 0.02 mol L^−1^ HCl or NaOH) and for pH = 4.0–8.0, buffer solutions were prepared by mixing the corresponding amounts 0.02 mol L^−1^ CH3COOH with 0.02 mol L^−1^ NaOH.

Other reagents were obtained from Fluka. All aqueous solutions were prepared with salts of the highest purity available (pure pro analysis) using freshly distilled deionized water (resistance 18.2 MΩ, Milli‐Q plus, Millipore, Austria).

##### Apparatus

The measurement of the electromotive force of the electrochemical cell: studied lead electrode–reference electrode Ag/AgCl (Metrohm 6.0750.100) was carried out at room temperature in a solution stirred with a magnetic stirrer by means of potentiometric system consisting of a 16‐channel data acquisition system (Lawson Labs. Inc., USA) and IBM PC computer. A pH meter Lab Star PH111 (Thermo Scientific Orion) and an Orion 81‐72 glass electrode were used for pH measurement. Sequential dilutions of stock solutions were performed using the 700 Dosino and 711 Liquino pump systems (Metrohm, Switzerland).

##### Preparation of the ISM

The ISM was prepared by weighing the individual membrane components in appropriate proportions (0.3 g in total) on an analytical balance and dissolving the resulting mixture in 3 mL of THF. The qualitative and quantitative composition of the HDES modified membrane was as follows: 1% lead ionophore IV, 33% PVC, 1%–12% HDES (MOA or TOA), and 53%–65% BBPA. A membrane without HDES with following composition: 1% lead ionophore IV, 33% PVC, 0.5% 1% KTPClPB, 65.5% BBPA was also prepared and used as a control system. The membrane components and organic solvent were mixed thoroughly and placed in an ultrasonic bath until the mixture was completely homogenized.

##### Preparation of ISEs

To prepare the electrode with liquid contact, the membrane cocktail was poured into a glass ring placed on a glass plate and allowed to evaporate the solvent. From the large disc of membrane thus obtained, small pieces of 5 mm diameter were cut and mounted in the Philips IS 561 electrode bodies, which were then filled with an internal solution of 1 × 10^−3^ mol L^−1^ Pb(NO_3_)_2_. Between measurements, electrodes were stored in the same solution.

For the best membrane composition (containing 5% MOA), electrode with solid contact was prepared using glassy carbon electrode (GCE) modified by CuONPs/MWCNT NC as inner electrode. It was prepared exactly according to the procedure described previously.^[^ ^30^ ^]^ In the beginning, GCE was thoroughly polished with Al_2_O_3_ powder, rinsed with water in an ultrasonic bath, and additionally immersed in THF and dried in air. Next, GCE was modified by NC by drop casting of 15 μL of NC suspension (2 mg NC/1 mL THF) onto the electrode surface and left for THF evaporation. After this, membrane was deposited on the modified GCE by drop casting 3 × 30 μL of membrane cocktail and left to dry for 24 h. Next day, the electrode was immersed in 1 × 10^−3^ mol L^−1^ Pb(NO_3_)_2_ solution for 12 h and then in conditioning solution with lower concentration. Conditioning solutions were used with lead concentrations of 1 × 10^−3^, 1 × 10^−5^, 1 × 10^−7^, and 1 × 10^−9^ mol L^−1^. The electrode was also stored in conditioning solution between measurements.

##### Sample Preparation

Tap water was analyzed without pretreatment, immediately after collection, just after the addition of ionic strength buffer solution (0.001 mol L^−1^ Mg(NO_3_)_2_) and acetate buffer solution pH 5.0 (0.01 mol L^−1^). Samples of river water were taken from the Jala River in the city of Tuzla. Samples were collected into clean polyethylene containers and filtered with a 0.45 μm Millipore membrane filter. Next, they were pretreated before being analyzed according to the procedure proposed by our group.^[^ ^41^, ^42^ ^]^ It was based on premixing the sample with Amberlite XAD‐7 resin for 2 min. During this time, organic substances were adsorbed onto the resin and did not disturb in potentiometric measurements.

## Conflict of Interest

The authors declare no conflict of interest.

## Author Contributions

**Cecylia Wardak**: conceptualization (lead); data curation (equal); investigation (lead); methodology (lead); supervision (lead); writing—original draft (equal); writing—review and editing (lead). **Malgorzata Grabarczyk**: conceptualization (supporting); investigation (equal); methodology (equal); writing—original draft (equal). **Mersiha Suljkanović**: data curation (supporting); investigation (equal); writing—original draft (supporting). **Jasmin Suljagić**: data curation (supporting); validation (equal); writing—original draft (supporting). **Magdalena Wardak**: data curation (supporting); investigation (supporting); validation (equal).

## Data Availability

The data that support the findings of this study are available from the corresponding author upon reasonable request.
